# Effects of α-Synuclein Overexpression in Transgenic *Caenorhabditis elegans* Strains

**DOI:** 10.2174/1871527311211080005

**Published:** 2012-12

**Authors:** Rakesh Bodhicharla, Archana Nagarajan, Jody Winter, Ademola Adenle, Aamir Nazir, Declan Brady, Kelly Vere, Jo Richens, Paul O’Shea, David R Bell, David de Pomerai

**Affiliations:** 1 Schools of Biology, University of Nottingham, University Park, Nottingham. NG7 2RD, UK;; 2 Schools of Clinical Sciences, University of Nottingham, University Park, Nottingham. NG7 2RD, UK;; 3 United Nations University Institute of Advanced Studies, 1-1-1- Minato Mirai, Nishi-ku, Yokohama 220-8502, Japan;; 4 Toxicology Division, Central Drug Research Institute, Mahatma Gandhi Road, Lucknow, 226 001, India;; 5 Directorate of Assessment B1, European Chemicals Agency, Annankatu 18, P.O. Box 400, FI-00120 Helsinki, Finland

**Keywords:** *Caenorhabditis elegans*, CFP and YFP reporters, FRET, protein aggregation, α-synuclein, transgenic strains.

## Abstract

The neural protein α-synuclein aggregates both *in vivo* and *in vitro* to form insoluble fibrils that are involved in Parkinson’s disease pathogenesis. We have generated α-synuclein/fluorescent-protein fusion constructs overexpressed in muscle cells of the nematode, *Caenorhabdtis elegans*. Green Fluorescent Protein (GFP) variants, Cerulean (C) or Venus (V), were fused to the C-terminus of human α-synuclein (S); the resultant fusion genes were designated SV and SC, plus a CV fusion as well as S, C and V singly. The aggregation behavior of the purified fusion proteins (expressed in *E. coli*) will be described elsewhere. These constructs were fused to a *C. elegans unc-54* myosin promoter, and integrated transgenic lines generated by microinjection, γ-irradiation, and outcrossing of fluorescent progeny. All transgenic lines expressing α-synuclein showed significant reductions (p < 0.05) in lifespan, motility and pharyngeal pumping, as compared to wild-type worms or lines expressing CFP and/or YFP only. We showed that CFP and YFP labels colocalised in granular inclusions throughout the body wall in transgenic lines expressing both SC and SV fusions (SC+SV), whereas SV+C worms displayed YFP-labelled inclusions on a diffuse CFP background. These findings implied that the α-synuclein moieties of these fusion proteins still aggregated together *in vivo*, whereas CFP or YFP moieties alone did not. This in turn suggested that Foerster Resonanace Energy Transfer (FRET) between CFP and YFP labels in α-synuclein aggregates could allow the extent of aggregation to be quantified. Accordingly, we also showed that net FRET signals increased 2-fold between L4 and adult SC+SV worms.

## INTRODUCTION

Certain proteins have a propensity to aggregate both *in vivo* and *in vitro*, leading ultimately to the formation of fibrillar aggregates (amyloid fibrils), which are a typical pathological feature of several important neurodegenerative diseases, such as Alzheimer’s and Parkinson’s [1, 2]. These large fibrillar aggregates may well be the end result rather than the cause of neuronal toxicity and cell death, since several recent studies implicate smaller protein oligomers as the key neurotoxic species [3]. The characteristic extra- or intra-cellular aggregates observed in Lewy bodies or in dopaminergic neurons within the substantia nigra in Parkinson’s disease patients [4] are principally composed of a small, natively unfolded neural protein called α-synuclein [5], which was first linked to the disease through two mutant variants (A53T or A30P) that cause autosomal dominantly inherited forms of the disease [6, 7]. However, generating suitable animal models of Parkinson’s disease has proved problematic in several respects [8], and several groups have resorted to simplified invertebrate models that mimic only certain aspects of the disease, e.g. in *Drosophila* [9, 10]. Typical among these are several transgenic *Caenorhabditis elegans* models overexpressing human α-synuclein in various cell types, e.g. in body-wall muscle [11, 12], pan-neuronally or else in dopaminergic neurons only [13]. The principal advantages of performing such studies in the *C. elegans* model [14] include its small size, transparency and anatomical simplicity, combined with its rapid development (3.5 days from egg to adult at 25°C), excellent genetics [15], reproduction predominantly by hermaphrodite self-fertilisation (allowing easy maintenance of mutant or transgenic stocks), straightforward production of transgenic lines [16, 17], complete genome sequence [18], and genome-wide RNA interference (RNAi) by feeding [19]. Transgenic GFP reporter strains of *C. elegans* allow direct monitoring of gene expression levels in living worms [20], a feature which has been exploited in strains expressing α-synuclein:GFP fusion proteins (such as the *unc-54*::α-synuclein:YFP construct in strain NL5901 [12, 21]).

The present study follows this well-established precedent, but with a novel twist. We have co-injected N2 worms with a mixture of *unc-54*::α-synuclein:CFP (α-synuclein fused at its C-terminus with the blue or Cyan variant of GFP; henceforward this fusion is designated SC) and *unc-54*::α-synuclein:YFP (α-synuclein similarly fused to the yellow or Venus variant of GFP; this fusion is designated SV), generating an integrated double transgenic strain that expresses both CFP and YFP fusion proteins (*unc-54*::SC+SV). *In vitro* aggregation studies using these same fusion proteins (expressed in *E. coli*) are being published separately. Since CFP emissions overlap with YFP excitation wavelengths, this combination of fluorescent tags allows the progress of α-synuclein aggregation to be monitored *via *Foerster Resonance Energy Transfer (FRET), whereby the donor CFP is excited at ~450 nm but emissions are read in the acceptor YFP range (~540 nm) [22]. Such FRET signals arise from donor CFP emissions exciting a nearby acceptor YFP molecule, which can only happen when the two fluorescent tags are in very close proximity (within about 70 Angstroms [22]) – effectively, within the same protein aggregate rather than free in cytosolic solution. However, cross-over between the CFP and YFP channels is inevitable (particularly for CFP signals in the YFP emission range), and this needs to be corrected for in any FRET-based assay (see Methods). To maximize expression of both fluorescent tags attached to α-synuclein, we chose to drive these constructs with the *unc-54* major myosin promoter, ensuring abundant expression of both fusion proteins within the body wall muscle. A series of other transgenic strains was also constructed using the same *unc-54* promoter, expressing CFP (C), YFP (V), α-synuclein (S) and various fusion proteins (SC and SV), either alone or in combination. This paper reports a preliminary characterization of these strains in terms of simple life history parameters such as developmental time, brood size, motility, pharyngeal pumping rate and lifespan, as well as monitoring α-synuclein aggregation during early adult life.

## MATERIALS AND METHODS

### Construction of Transgenic Strains

N2 and NL5901 (essentially an integrated α-synuclein:YFP fusion construct driven by the *unc-54 *promoter [12]) strains of *C. elegans* were obtained from the Caenorhabditis Genetics Center (University of Minnesota, USA; funded by the NIH National Center for Research Resources). All transgenes generated in this study are listed in Table **1**, and the design strategy is outlined in Fig. (**1**). Essentially, the human α-synuclein coding sequence (here designated S) [23] was fused at its C-terminus to either Cerulean (cyan CFP; designated C) [24] or Venus (yellow YFP, designated V) [25] variants of GFP, with no added linker apart from 2 amino acids (phe-glu) introduced by the cloning site. Transgenic worms carrying the desired gene of interest were produced by micro-injection of recombinant DNA (100 ng μl^-1^ of each plasmid; < 1 ng per worm) into the worm gonad, where they were transferred as extra-chromosomal arrays to unfertilized eggs. The fluorescent protein tags served as markers here, indicating which worms were transgenic. Transmitting lines for the various transgenes of interest were obtained and maintained by selection of fluorescent worms. Strains with stable chromosomally integrated transgenes were obtained by γ-irradiation at 2000-4000 rads (gamma cell unit with a ^137^Caesium source; Nordion International Inc). Progeny of irradiated worms were screened for 100% transmission of transgenes and then outcrossed 5 times with wild-type N2 to remove deleterious mutations elsewhere in the genome. These procedures followed standard Wormbook protocols for generating integrated transgenic lines of *C. elegans* [26]. Worms were cultured on standard Nematode Growth Medium (NGM) agar plates using a *lac*-deleted strain of *E. coli* (P90C) as food source, as described previously [27]; all worm washing and handling used K medium (53 mM NaCl, 32 mM KCl [28]) throughout.

For initial cloning of fusion genes, Cerulean (CFP, C), Venus (YFP, V) and synuclein (S) were PCR amplified and cloned into pGEMT Easy with restriction sites added to the ends as follows:

- NcoI-Cerulean-SacI
- NcoI-Venus-SacI
- NcoI-Synuclein-SacI
- NcoI-Cerulean-Csp45I
- NcoI-Synuclein-Csp45I
- Csp45I-Cerulean-SacI
- Csp45I-Venus-SacI

Clones were sequenced to exclude the possibility of PCR-induced mutations. Cloned genes were then excised from pGEMT Easy using the restriction enzymes listed above, and ligated into pET-30a either singly or in pairs. pET-30a was double digested with NcoI and SacI prior to ligation. For double ligations, NcoI-gene-Csp45I and Csp45I-gene-SacI were simultaneously ligated into pET-30a to produce gene fusions. They were further subcloned into pPD 30.38 (Addgene Fire lab vector). The following products were subcloned into pPD 30.38 in this way:

- Cerulean     C
- Venus      V
- Synuclein     S
- Cerulean-Venus  CV
- Synuclein-Cerulean SC
- Synuclein-Venus  SV

### Egg to Adult Development Time

The egg to adult development time of worms was monitored by picking individual hermaphrodites at the late L4 stage on to separate NGM agar plates. On reaching the adult stage, they were allowed to lay eggs for 2 hours, then the adults were removed. Eggs were allowed to develop at 20ºC until the larvae reached adulthood and began to lay eggs. The developmental time for each worm was calculated from egg to egg-laying adult.

### Brood Size

L4 hermaphrodites of the desired genotype were transferred individually onto NGM agar plates at 20°C, and transferred to fresh plates at 24 hour intervals. The progeny were counted at late larval to adult stages by flaming worms one by one. An embryo was scored as being dead if the egg had not hatched after 24 hours at 20°C. The brood size for each animal was calculated as the sum of both hatched and non-hatched progeny.

### Pharyngeal Pumping Rate

Pharyngeal pumping rates were determined by picking 10 adult worms from the bacterial lawn on an NGM agar plate and transferring them onto a bacteria-free plate, since it was difficult to observe pharyngeal pumping on food-containing agar plates as worms tend to hide under the bacteria. Pharyngeal pumping was observed for each of the 10 worms for 1 minute at room temperature (~ 20°C) using a dissection microscope (Olympus Model SZX12).

### Locomotion Rate

Locomotion rates were measured by picking individual adult worms from an NGM agar plate with bacteria and transferring them onto a bacteria-free plate. The number of bends performed by each worm over a 30 second interval was then determined. 10 such worms were measured for each strain at room temperature (~ 20°C), using a dissection microscope as above.

### Lifespan

The lifespan of worms was determined by picking individual hermaphrodites at the late L4 stage onto separate plates. They were left for 2 hours to produce eggs, then the adults were removed. The eggs were allowed to develop at 20ºC until larvae reached the L4 stage, when they were transferred onto a separate plate and incubated overnight at 20ºC. Next day the adults were transferred onto a new plate leaving their offspring behind on the old plate. The same process was repeated until the worms stopped producing offspring (usually 3-4 days). The adult worms were allowed to grow on at 20ºC, and were monitored daily by tapping them on the head. Animals were considered dead if no movement was observed following repeated probing. Once worms were identified as dead, their lifespan was calculated from egg until death.

### FRET Analysis of Unc-54 :: SC+SV and SV Worms

SC+SV and SV worms were grown on 14 cm NGM plates for 4 days at 15ºC and synchronized by L1 filtration using a 5µm mesh filter [29]. This crude method of synchronization was chosen in preference to the standard method of egg isolation by bleaching, in order to avoid possible confounding effects on α-synuclein aggregation caused by treatment with toxic bleach (NaOCl). To monitor FRET signals during ageing, synchronized L4 larvae of both SC+SV and SV (NL5901 [12]) strains were washed repeatedly with K medium [28] and worm numbers counted. Worm suspensions were diluted as necessary to equalize worm numbers between the strains and time points compared, and were stirred continuously during dispensing [20]. At least 4 replicate 300 µl aliquots (each containing 1000 to 1500 worms) were placed in separate U-bottomed wells in 96-well non-fluorescent black plastic plates (Nunc Ltd, from Fisher Scientific, Loughborough, UK) and kept on ice for 10-15 min to allow worms to settle at the bottom of each well. Plates were read in a Perkin-Elmer Victor 1420 Multi-Label Reader (< 2 min per plate), using narrow bandpass filters at 430 nm for CFP excitation and at 530 nm for YFP emission (total FRET). For determinations of CFP, excitation was at 430 nm and emissions read at 486 nm; for YFP determinations, excitation was at 510 nm and emissions read at 530 nm. All three fluorescence readings were taken in rapid succession for each well. At the conclusion of the FRET measurements, worms of both strains were collected by centrifugation and cultured on standard NGM agar plates, with L1 filtration [29] on each of 4 successive days so as to remove offspring, returning the adults to the culture plates each time. A second set of FRET measurements was undertaken for both strains on day 6 of adult life. We have obtained essentially identical results (data not shown) using non-reproducing cultures of worms maintained from L4 onwards on NGM plates containing 200 µM fluorodeoxyuridine (FUdR) to maintain synchronicity without reproduction [30]. Based on the cross-over between CFP and YFP channels measured for worms expressing *unc-54*::C (non-integrated) or *unc-54*::SV (NL5901, integrated; [12]), a corrected net FRET value was calculated according to the following formula:

Corrected net FRET = total FRET - [(0.45 × CFP reading) + (0.07 × YFP reading)].

For CFP-only worms (C), the total FRET signal was 45% of the measured CFP fluorescence. Although this *unc-54*::C strain is non-integrated, its transgene is transmitted with high efficiency to ~80% of progeny. Worms were grown up for 1 generation only from picked fluorescent L4 larvae.

For YFP-only worms (NL5901), the total FRET signal was only 7% of the measured YFP fluorescence. Note that our own *unc-54*::SV strain is non-integrated, but like *unc-54*::C (above) transmits its transgene to ~80% of its progeny. However, strain NL5901 was used for Figs. (**4-6**).

FRET signals are given in Relative Fluorescence Units, which are directly comparable only within the same strain and run. However, percentage changes relative to an L4 baseline are robust to changes in worm number, and are very reproducible from one run to another (data not shown).

### Confocal Microscopy of SC+SV and SV+C Worms

For confocal microscopy, worms were removed from the microplate wells and immobilized in a final concentration of 10 mM sodium azide and 50% (v/v) glycerol. After mounting on slides and sealing the coverslips with nail polish, confocal microscopy was performed using a Leica TCS SP2 Confocal Laser Scanning Microscope; CFP excitation used a laser set at 458 nm with an emission range from 465 to 600 nm, whereas YFP excitation used a 514 nm laser with an emission range from 525 to 600 nm. YFP emissions (525-600 nm) were also examined after CFP excitation at 458 nm, providing a FRET image with no detectable cross-over from the CFP channel (see Fig. **5c**). YFP fluorescence was also examined in *unc-54*::SC+SV worms both at day 1 and day 6 adult stages; controls included non-integrated CFP-expressing *unc-54*::C and *unc-54*::SV+C strains.

### Statistical Analysis

Mean data from all the assays (except life span and FRET) were analysed using one-way ANOVA followed by Dunnett’s multiple comparisons test against wild-type N2 as control. Since lifespan was calculated as percentage survival, the data were analysed using a Mantel-Cox log rank test. For the FRET analysis, unpaired t-tests were used.

## RESULTS

### Egg to Adult Development Time

**Fig. (2):**
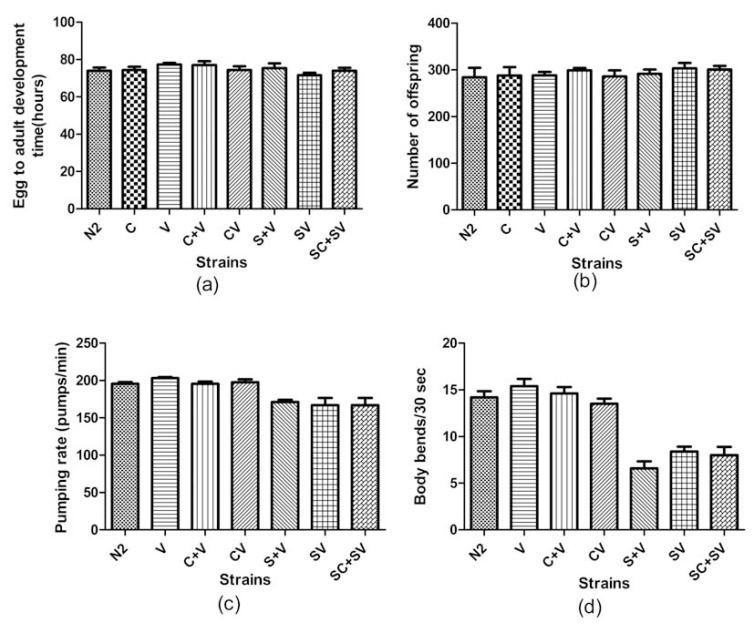
(**a**) Egg to adult development time, (**b**) Brood size, (**c**) Pharyngeal pumping rate and (**d**) Locomotion rate for the various transgenic worm strains tested. Error bars show the standard error of the mean. All trangenic strains were integrated lines, apart from C and SV which were both non-integrated lines showing high transmission (~80%) of the transgene to progeny.

All strains took between 70 and 80 hours to reach adulthood (Fig. **2a**). One-way ANOVA revealed no significant effect of strain (*F* = 1.031, *p* = 0.448; Table **2**), and Dunnett‘s multiple comparison test against N2 controls confirmed that none of the transgenic strains tested differed from N2 in terms of developmental time (*p* > 0.05).

### Brood Size

One-way ANOVA showed no statistical difference between strains for brood size (*F* = 0.326, *p* = 0.938, Table **2**), which for all strains was close to 300, confirming that they were all normal [31]. There was also no statistically significant difference between transgenic strains (with or without α-synuclein) and N2 controls (*p *> 0.05, Dunnett’s test) (Fig. **2b**).

### Pharyngeal Pumping Rate

Transgenic α-synuclein-expressing worms showed slower pharyngeal pumping rates when compared with the transgenic strains lacking α-synuclein or with wild type N2 worms. One-way ANOVA showed a significant strain effect (*F* = 8.06, *p* < 0.0001, Table **2**). Using Dunnett‘s multiple comparisons test, the transgenic α-synuclein-expressing worms (S+V, SV and SC+SV) were found to be significantly different (*p* < 0.05, *p* < 0.001 and *p* < 0.001, respectively) from the control wild-type N2 worms, whereas the transgenic strains lacking α-synuclein (V, CV and C+V) were not (*p *> 0.05 in all cases) (Fig. **2c**).

### Locomotion Rate

One-way ANOVA revealed a significant effect of strain on locomotion rate (*F* = 21.76, *p* < 0.0001, Table **2**). Transgenic α-synuclein-expressing worms moved more slowly than transgenic strains lacking α-synuclein, or wild type N2 worms. Using Dunnett‘s multiple comparisons test, the transgenic α-synuclein-expressing worms (S+V, SV and SC+SV) were statistically significantly different (*p* < 0.001 for all 3 strains) from control wild-type N2 worms, whereas the strains lacking α-synuclein (V, CV and C+V) were not (*p *> 0.05) (Fig. **2d**).

### Lifespan

As shown in Fig. (**3a**), the 3 lines expressing one or both fluorescent markers but lacking α-synuclein (V, CV and C+V) showed only a slight (1-2 days) reduction in lifespan compared to N2 worms at 20 °C, which was non-significant (*p* = 0.08, 0.137 and 0.555, respectively; Mantel-Cox test). This is consistent with other studies, which showed that expression of GFP reporters does not in itself affect lifespan significantly [32]. However, as shown in Fig. (**3b**), the 3 lines expressing α-synuclein (S+V, SV and SC+SV) all showed a marked decrease of ~5 days in lifespan as compared to N2 (*p* < 0.0001 for all 3 strains; Mantel-Cox test). Thus over-expression of α-synuclein in these worms caused significant toxicity and reduced lifespan, as well as the reduced motility and pharyngeal pumping noted earlier (Fig. **2c**, **d**).

### FRET Analysis of SC+SV Worms

**Fig. (4):**
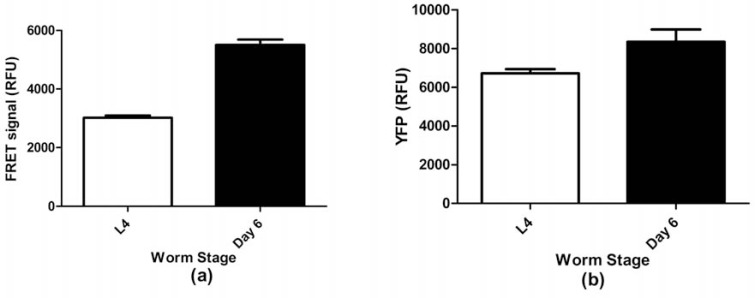
(**a**) Corrected net FRET signal of L4 and day 6 SC+SV worms and (**b**) YFP signal of L4 and day 6 NL5901 (SV) worms. Error bars show the standard error of the mean.

**Fig. (5):**
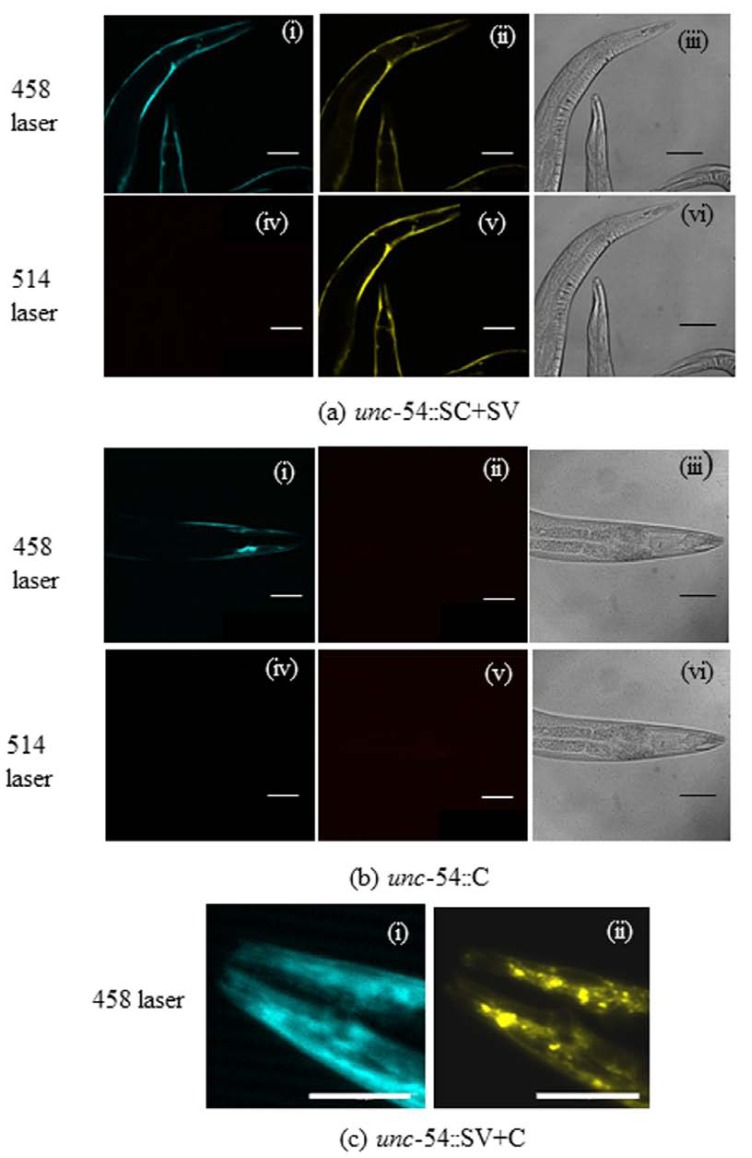
**Confocal Microscopy**: (**a**) *unc*-54::SC+SV worms (I) showing FRET signal with CFP excitation, (**b**) control *unc*-54::C worms (NI) showing the absence of FRET signal under identical conditions and (**c**) the head region of a young adult *unc-54*::SV+C worm (NI) showing diffuse CFP but aggregated YFP. The excitation and emission wavelengths used were 458/485 nm for CFP, and 514/545 nm for YFP. All photographs used the same gain settings and exposure times; scale bars =100µm. Each part [(**a**), (**b**) and (**c**)] shows images of the same worm, using CFP fluorescence [(**a**)i, (**b**)i, (**c**)i], FRET [(**a**)ii, **b**(ii)], CFP fluorescence with YFP excitation [(**a**)iv, (**b**)iv] or YFP fluorescence [(**a**)v, (**b**)v, (**c**)ii]. Differential interference contrast (DIC) images of the same worms are shown in panels (**a**)iii, (**a**)vi, (**b**)iii and (**b**)vi. I = integrated strain. NI = non-integrated strain.

The corrected net FRET data for doubly transgenic *unc-54*::SC + *unc-54*::SV (henceforward *unc-54*::SC+SV) worms were analysed using unpaired t-tests, which showed that net FRET significantly increased with age (*t* = 12.45, *p* < 0.0001). We simultaneously measured reporter gene expression changes using *unc-54*::SV (NL5901 [12]) worms, and found some increase in YFP expression also (*t* = 2.87, *p* = 0.02). However, the increase in net FRET was proportionately much larger than the increase in *unc-54* reporter expression (82% increase versus 31%), thus indicating an apparent increase in α-synuclein aggregation with age (Fig. **4a**, **b**).

### Confocal Microscopy

Confocal microscopy of SC+SV worms with excitation at 458 nm showed a strong CFP signal but also robust FRET (Fig. (**5a**) i and ii). When excited at 514 nm, the worms showed no CFP signal but strong YFP fluorescence (as expected; Fig. (**5a**) iv and v). Worms expressing only CFP (*unc54::*C) showed fluorescence only in the CFP emission range, but no FRET (Fig. (**5b**) i and ii); moreover, no fluorescence was apparent in either channel using YFP excitation at 514 nm (Fig. (**5b**) iv and v). This control confirmed that the YFP signal seen with CFP excitation in SC+SV worms (Fig. (**5a**) ii) is genuine FRET rather than cross-over from CFP emissions into the YFP emission range. Cross-over from YFP signals into the CFP channel was minimal (see Methods). In Fig. (**5c**), the head of an *unc-54*::SV+C worm (non-integrated line) showed strongly granular YFP but diffuse expression for CFP (compare Fig. (**5c**) i and ii), suggesting that the α-synuclein moiety is essential for aggregation into these granular inclusions.

**Fig. (6):**
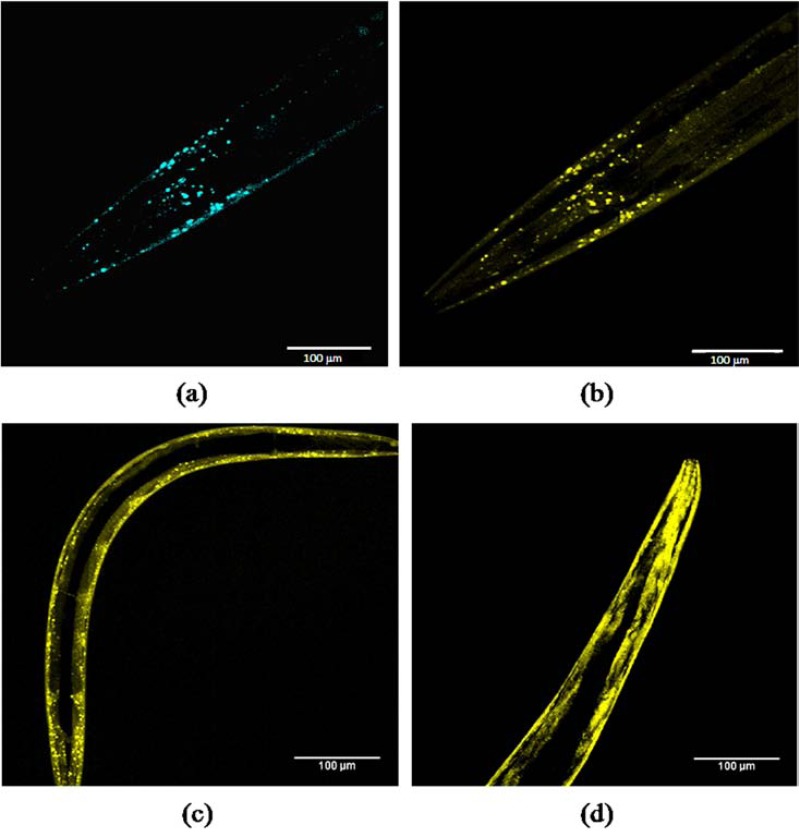
**Confocal microscopy of *unc*-54:: SC+SV L4 and adult worms (I).** Body wall muscles of worms with Z-stacking for CFP (part a) or YFP (parts b-d): (a) L4 larval head imaged for CFP (458 nm laser, 485 nm emission filter); (b) the same L4 head region imaged for YFP (514 nm laser, 545 nm emission filter); (c) 1-day old adult showing small YFP-labelled granules; (d) 6-day adult showing larger and more intense or merged YFP-labelled granules. The YFP excitation and emission wavelengths for parts c and d were the same as for part b. All images used the same exposure time and gain settings; scale bar =100µm. I = integrated strain.

Confocal microscopy of SC+SV worms with Z-stacking showed that the distribution of granules for both CFP (Fig. **6a**) and YFP (Fig. **6b**) was virtually coincident, using the same excitation lasers and emission filters as for Fig. (**5**) (i.e. with virtually no cross-over). Thus both SC and SV transgenes were abundantly expressed, and even small granules contained both fluorescent tags. A comparison between 1-day and 6-day adult worms suggested an increase in these granular inclusions with age. At 1 day (Fig. **6c**), the granules of aggregated α-synuclein were mostly small, but these increased in size and intensity or merged by day 6 of adult life (Fig. **6d**). These findings reinforced the conclusion from the FRET measurements in Fig. (**4**), indicating that α-synuclein aggregation increased over time during the first 6 days of adult life.

## DISCUSSION

Previous transgenic *C. elegans* models involving *unc-54*-driven GFP- or YFP-tagged α-synuclein constructs expressed in *C. elegans* body-wall muscle have been used mainly for hypothesis-driven [11] or genome-wide [12] RNAi screening to identify genetic modifiers of α-synuclein aggregation. These have relied essentially on image analysis to identify genes whose inactivation increases (or decreases) the number/size/intensity of fluorescent granules within the test worms. Inevitably, there is an element of interpretation here, which might result in some false positives or negatives; moreover, results may be influenced by factors that modify transgene expression levels or cause small aggregates to coalesce. Lastly, the toxic species of aggregated α-synuclein are most likely small oligomers rather than the larger granular inclusions which are screened for. In all of these respects, there are clear advantages to be gained from developing a FRET-based quantitative assay using doubly transgenic SC+SV worms expressing both CFP and YFP-tagged α-synucleins in the same cells (body-wall muscle, driven by the *unc-54 *promoter). Bulk fluorescence changes reflecting altered transgene expression or the spatial distribution of fluorescence can be controlled for by running parallel assays on the freely available NL5901 *unc-54*::SV strain [12]. Moreover, increases in FRET signal might be detectable during α-synuclein oligomerisation without any accompanying increase in fluorescent granule formation, since FRET could occur within small oligomers so long as both CFP and YFP fluorophores are present together in close proximity. It is reassuring to note that *in vitro* aggregation studies using YFP-tagged α-synuclein demonstrate that aggregation is not greatly impeded by the presence of the larger YFP moiety [21]; furthermore, the *in vivo* aggregation of this same fusion protein expressed in NL5901 worms is greatly exacerbated by RNAi against both *hsp-70* and *hip-1* [33]. The limitations of currently available *C. elegans* models and advantages of our *unc-54*::SC+SV strain are summarized in Table **3**.

This study reports the development of an *unc-54*::SC+SV strain which fulfils most of these aims. Broadly speaking, the overexpressed α-synuclein fusion proteins formed fluorescent granular inclusions throughout the body-wall muscle, similar to those reported previously for the *unc-54*::SV strain NL5901 [12]. These granules increased in size and intensity in older worms, consistent with recent reports that widespread protein aggregation accompanies normal ageing in *C. elegans* [34]. There were also clear adverse effects of α-synuclein overexpression on several life-history parameters, including lifespan, motility and pharyngeal pumping rate, though not on brood size or developmental time. A transgenic strain overexpressing wild-type or mutant α-synuclein only in neurons (using the *aex-3* or *dat-1* promoter) showed an increased lifespan partly attributable to caloric restriction as a result of decreased pharyngeal pumping, as well as decreased egg-laying [35]. In our *unc-54*::SC+SV strain, the reduction in pharyngeal pumping (< 20%) was much less than the reduction in overall motility (~50%), consistent with the fact that the SV and SC transgenes are expressed in body-wall but not in pharyngeal muscle cells. Overall, any effect of reduced feeding on lifespan is likely to be outweighed by other detrimental effects of α-synuclein overexpression in our SC+SV and other S-expressing worms.

Notably, the adverse effects on life-history traits reported in this study were confined to those strains expressing an α-synuclein construct, whereas no significant effects were observable in strains similarly overexpressing the CFP or YFP fluorescent markers. This strongly suggests that the fluorescent granules in *unc-54*::SC+SV worms resulted from aggregation of the attached α-synuclein moieties. Moreover, in an *unc-54*::SV+C strain, the CFP fluorescence remained diffuse while the YFP was strongly granular, again suggesting that aggregation was dependent on α-synuclein, and did not simply result from overexpressed proteins aggregating randomly.

It might be thought preferable to develop *C. elegans* models expressing such fluorescent-tagged α-synuclein constructs either pan-neuronally or even in the dopaminergic neurons only [13]. Although we have prepared a similar set of fusion constructs attached to the pan-neuronal *aex-3* promoter, we chose to start by deliberately overexpressing these fusion proteins throughout the body-wall muscle, so as to generate strong fluorescent signals where genuine FRET could be distinguished both from background noise and from the inevitable cross-over between CFP and YFP channels. We believe that our *unc-54*::SC+SV strain will prove useful for identifying environmental risk factors that might contribute to the development of human Parkinson’s disease – particularly since the majority of cases are idiopathic rather than genetic [6, 7] in origin. Moreover, our SC+SV strain can readily be used for high-throughput screening (through simple quantitative FRET measurements), allowing mixtures of agents to be tested (Table **3**). The interactions between environmental and genetic (explored *via *RNAi) factors can also be studied using this strain, with positive hits confirmed through careful comparison against control strains (e.g. NL5901; Fig. **4**). Lastly, given that fluorescent granules are already apparent in L4 larvae and increase during adult life (Fig. **6**), this strain readily lends itself to the screening of anti-aggregation drug candidates. However, because of the muscle site of transgene expression, this model is unlikely to respond to drug interventions targeting dopaminergic functions specifically, except perhaps through general effects on oxidative stress.

## Figures and Tables

**Fig. (1):**
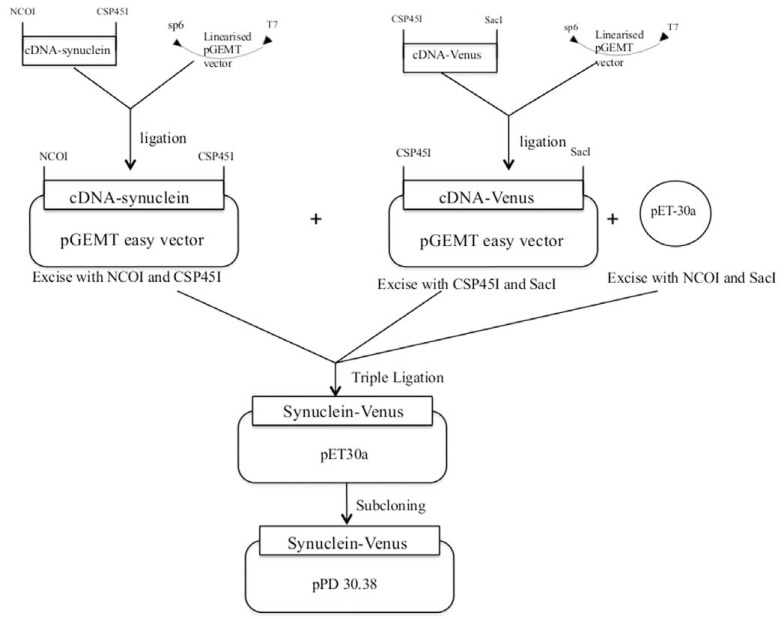
Schematic representation of the cloning and sub-cloning of α-synuclein fused with Venus (YFP). All other fusion transgenes were created in a similar fashion.

**Fig. (3):**
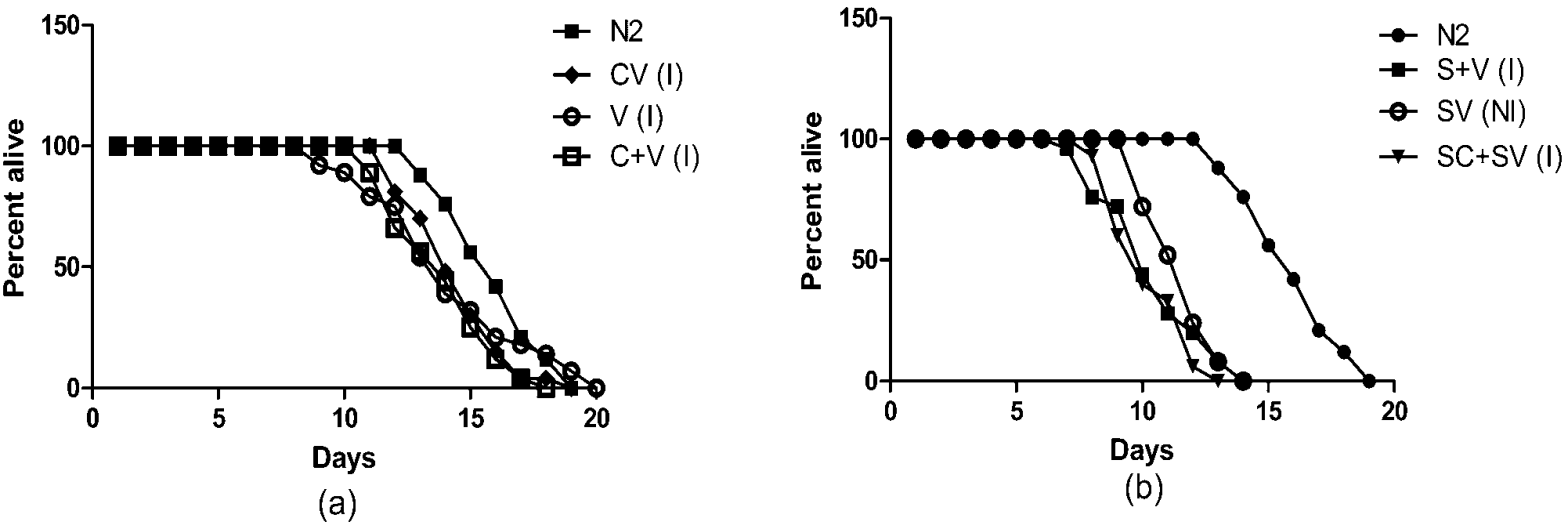
Lifespans of (**a**) non-synuclein and (**b**) α-synuclein expressing transgenic worms, calculated as the percentage left alive on each day. I = integrated strain; NI = non-integrated SV strain (only fluorescent worms were selected for the lifespan analysis).

**Table 1.** Transgenes and Gene Constructs

No.	Transgene and Integration Status	Gene Construct with Restriction Sites
1	Cerulean (C) Non-integrated	NcoI-Cerulean-SacI
2	Venus (V) Integrated	NcoI-Venus-SacI
3	Cerulean + Venus (C+V) Integrated	NcoI-Cerulean-SacI + NcoI-Venus-SacI
4	Cerulean-Venus Integrated	NcoI-Cerulean-Csp45I/Csp45I-Venus-SacI
5	Synuclein + Venus Integrated	NcoI-Synuclein-SacI/NcoI-Venus-SacI
6	Synuclein-Venus (SV) Non-integrated*	NcoI-Synuclein-Csp45I + Csp45I-Venus-SacI
7	Synuclein-Venus + Synuclein-Cerulean Integrated	NcoI-Synuclein-Csp45I/Csp45I-Venus-SacI + NcoI-Synuclein-Csp45I/Csp45I-Cerulean-SacI

List of all the transgenes expressed in *C. elegans* using the *unc-54* promoter. Restriction enzymes were added to cut these gene constructs and various combinations used for construction of gene-fusions as indicated in the table. The genes were cloned into pGEMT-easy plasmid and sub-cloned into pET30a and subsequently into pPD30.38 (Fire lab vector, Addgene; http://www.addgene.org/firelab/ ) incorporating the *unc-54* promoter site. A slash (/) designates fusion of the sequences indicated, whereas a plus sign (+) indicates a mixture of both sequences. The integration status of each strain is indicated in the second column.

* Although our own *unc-54*::SV strain was non-integrated, a very similar construct is fully integrated in strain NL5901.

**Table 2.** 

Trait	MS	*df*	*F*	*p*	N2 Control (Mean ± SD)
Egg to adult development time	9.881	**7**	1.031	0.448	74 ± 3 hours
Brood size	320.1	7	0.326	0.938	284.2 ± 45.7 eggs
Pharyngeal pumping rate	1305	6	8.061	< 0.0001	195.8 ± 5.2 pumps/min
Locomotion rate	85.28	6	21.76	< 0.0001	14.2 ± 1.5 bends/min

Summary of One-Way ANOVA tests performed on egg to adult development time, brood size, pharyngeal pumping rate and locomotion rate, where strain was treated as a fixed factor. Units of analysis and mean N2 control values (± SD) are shown in the final column

**Table 3.** Summary of Advantages and Drawbacks of *C. elegans* Models for Parkinson’s Disease

	SC+SV	NL 5901	P_dat-1 _: WT and A53T and P_aex-3_:WT and A53T
Expression	Body-wall muscle cells (*unc-54*)	Body-wall muscle cells (*unc-54*)	Either pan-neuronally (*aex-3*) or only in dopaminergic neurons (*dat-1*)
Gene expression and RNAi studies?	Yes	Yes	Yes
Strong signal (reflecting higher expression levels)?	Yes	Yes	No (better for *aex-3* than *dat-1*, which is expressed in 5 dopaminergic cells)
Relevance to human Parkinson’s disease?	Limited to synuclein aggregation	Limited to synuclein aggregation	Yes (construct expressed in neuronal cells).
Quantification of aggregation?	Yes (using FRET)	Indirect (requires image analysis)	Indirect (requires image analysis)
High-throughput?	Yes (using FRET)	No (requires image analysis)	No (requires image analysis)

## ABBREVIATIONS

A30P and A53T: = Mutant versions of Synuclein leading to familial Parkinson’s disease.
*aex-3*: = *C. elegans* gene expressed pan-neuronally
C: = Cerulean (CFP)
CFP: = Cyan (blue) Fluorescent Protein
CGC: = *Caenorhabditis* Genetics Center (University of Minnesota)
C-terminus: = carboxy terminus of protein
CV: = CFP sequence fused at its carboxy terminus to YFP sequence
*dat-1*: = *C. elegans* gene expressed in dopaminergic neurons only
FRET: = Foerster Resonance Energy Transfer
FUdR: = Fluorodeoxyuridine
GFP: = Green Fluorescent Protein
*hsp-70* and *hip-1*: = Genes implicated in dealing with protein aggregates *in C. elegans*
I: = Integrated transgenic strain
K medium: = *C. elegans* saline (53 mM NaCl, 32 mM KCl)
L1 to L4: = larval stages of *C. elegans*
N2: = Wild-type (Bristol) strain of *C. elegans*
NGM: = Nematode Growth Medium
NI: = Non-Integrated transgenic strain
NL5901: = An *unc-54*::SV transgenic strain available from CGC
PCR: = Polymerase Chain Reaction
phe-glu: = Dipeptide sequence (phenylalanine followed by glutamic acid) introduced by cloning
RFU: = Relative Fluorescence Units
RNAi: = RNA interference
S: = Human Synuclein coding sequence
SD: = Standard Deviation
SEM: = Standard Error of Mean
SC: = Human Synuclein coding sequence fused at its C terminus to CFP
SV: = Human Synuclein coding sequence fused at its C terminus to YFP
*unc-54*: = Promoter sequence from major muscle myosin gene in *C. elegans*
(*unc-54*::)C: = *C. elegans* carrying a (non-integrated) *unc-54*-regulated CFP (C) transgene
(*unc-54*::)C+V: = *C. elegans* carrying both *unc-54*-regulated CFP and *unc-54*-regulated YFP transgenes
(*unc-54*::)S+V: = C. elegans carrying both *unc-54*-regulated S and *unc-54*-regulated YFP transgenes
(*unc-54*::)SC+SV: = *C. elegans* carrying both *unc-54*-regulated SC and *unc-54*-regulated SV transgenes
(*unc-54*::)SV: = *C. elegans* carrying an *unc-54*-regulated SV transgene
(*unc-54*::)SV+C: = *C. elegans* carrying both *unc-54*-regulated SV and unc-54-regulated CFP transgenes
V: = Venus (YFP)
YFP: = Yellow Fluorescent Protein
